# Effective heart-sparing whole lung irradiation using volumetric modulated arc therapy: a case report

**DOI:** 10.1186/s13256-019-2209-2

**Published:** 2019-09-02

**Authors:** Gen Suzuki, Toshiyuki Ogata, Norihiro Aibe, Hideya Yamazaki, Shigeki Yagyu, Tomoko Iehara, Hajime Hosoi, Kei Yamada

**Affiliations:** 1 Department of Radiology, Kyoto Prefectural University Graduate School of Medical Science, 465 Kajiicho Kawaramachi Hirokoji, Kamigyo-ku, Kyoto, 602-8566 Japan; 2 Department of Pediatrics, Kyoto Prefectural University Graduate School of Medical Science, 465 Kajiicho Kawaramachi Hirokoji, Kamigyo-ku, Kyoto, 602-8566 Japan

**Keywords:** Volumetric modulated arc therapy, Whole lung irradiation, Wilms’ tumor, IMRT, VMAT

## Abstract

**Background:**

Late cardiovascular disease-related adverse events are one of the most common causes of premature mortality among long-term survivors of childhood cancer. As it is difficult to reduce the heart dose with traditional anteroposterior–posteroanterior field whole lung irradiation for pulmonary metastasis, improved radiation techniques are highly desirable. We report a case treated with whole lung irradiation using volumetric modulated arc therapy.

**Case presentation:**

A 3-year-old Japanese girl with pulmonary metastases of Wilms’ tumor received 12 Gy in 8 fractions of whole lung irradiation using volumetric modulated arc therapy. The treatment was well tolerated, and the course was completed as planned without any toxicity. We found statistically significant reduced volumetric modulated arc therapy irradiation doses to organs at risk relative to those of the standard anteroposterior–posteroanterior field technique. The mean heart dose was 8.5 Gy for volumetric modulated arc therapy and 12.3 Gy for the anteroposterior–posteroanterior field. The doses to liver and thyroid were also more favorable with volumetric modulated arc therapy than with the anteroposterior–posteroanterior field technique. We confirmed the dosimetric advantages of volumetric modulated arc therapy over anteroposterior–posteroanterior field in whole lung irradiation in terms of superior normal organ protection.

**Conclusions:**

Effective heart sparing is possible for whole lung irradiation using volumetric modulated arc therapy. Large-scale studies using standardized procedures should be conducted to validate our results.

## Background

Wilms’ tumor (WT) is the most common renal neoplasm of childhood. Radiotherapy has historically had an important role as one of the main therapeutic tools for WT. The prevalence of adverse events in long-term survivors of WT is high, and an increased prevalence of cardiovascular events after chest irradiation has been reported [1]. Whole lung irradiation (WLI) has been widely used in the management of pulmonary metastasis, a concept that was introduced approximately half a century ago [2]. In the National Wilms Tumor Study Group (NWTS)-3 and NWTS-4, the 4-year event-free survival (EFS) and overall survival (OS) in patients with computed tomography (CT)-only lung metastasis was 89% and 91% after WLI compared with 80% and 85% with chemotherapy alone [3]. A UK Children’s Cancer Study Group report showed that patients with a chest X-ray with positive favorable histology for Wilms tumors had improved EFS and OS (79% and 85%) after WLI compared with chemotherapy alone (53% and 73%) [4]. WLI has traditionally been performed by using the anteroposterior–posteroanterior (AP–PA) field technique; however, one of the biggest drawbacks of this technique is the inability to reduce the high-dose volumes to the heart.

The use of volumetric modulated arc therapy (VMAT) has increased steadily in external beam radiotherapy. VMAT is a radiation technique that delivers highly conformal dose distributions through the complete rotation (360°) and speed variation of the linear accelerator gantry. This technique can be considered an extension to dynamic multi-leaf collimator intensity-modulated radiation therapy (IMRT). Compared with fixed-field IMRT, VMAT is capable of creating analogous or better dose distributions while reducing treatment time and monitor units by half [5]. To the best of our knowledge, the only reported case involving use of VMAT for WLI is a study by Papachristofilou *et al.* [6]. In their case, the radiation dose was much higher (18 Gy) than the dose generally used (12–15 Gy), which made it difficult to serve as a precise clinical reference. We report a case involving a patient who was administered a standard dose (12 Gy) of WLI using VMAT and present a subsequent dosimetry comparison of VMAT with the standard AP–PA field technique.

## Case presentation

**Fig. 1:**
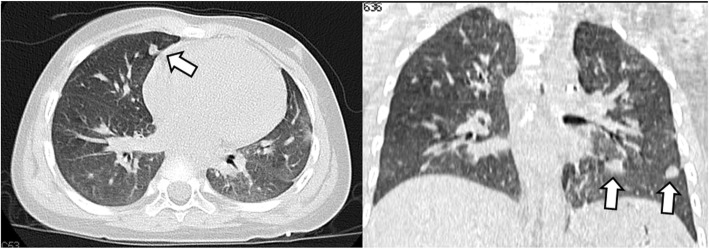
Computed tomography image of the chest showing multiple lung nodules (*arrows*)

A 3-year-old Japanese girl with pulmonary metastases of WT was referred to our department for WLI and adjuvant chemotherapy as per standard of care [7]. She had a past medical history of patent ductus arteriosus (PDA) and underwent ligation of PDA at the age of 9 months. She was receiving orally administered furosemide and spironolactone for 6 months after surgery. After surgery, she had normal growth. Her social and family history was unremarkable. Her environmental history revealed no abnormalities. She was diagnosed as having clinical stage I WT of the right kidney at the age of 2.5 years. She received a complete resection of the primary tumor and adjuvant chemotherapy with actinomycin D and vincristine. Since then, she had been taking sulfamethoxazole trimethoprim 500 mg twice daily. Multiple bilateral lung metastases were detected by CT images 1 month after the adjuvant chemotherapy (Fig. 1). A lung biopsy revealed metastatic disease and the response to chemotherapy was determined to be inadequate. Therefore, this patient was referred to our department for WLI and adjuvant chemotherapy as per standard of care [7]. Her physical examination on admission revealed blood pressure of 110/64 mmHg, pulse rate of 120 beats per minute, and temperature of 37.0 °C. Auscultation revealed normal heart sounds and clear lungs. The result of her cardiovascular examination was normal. The rest of her clinical examination was unremarkable. Her laboratory findings were as follows: hemoglobin 11.0 g/dL (normal range, 11.6–14.8 g/dL); hematocrit 33.1% (normal range, 35.1–44.4%); white blood cell count of 5.4 × 10^3^/mm^3^ (normal range, 3.3–8.6 × 103/mm^3^) with 32.4% neutrophils, 56.1% lymphocytes, 0.37% monocytes, and 4.4% eosinophils; platelet count 293 × 10^3^/mm^3^ (normal range, 158–348 × 103/mm^3^); sodium 139 mmol/L (normal range, 138–145 mmol/L); potassium 3.9 mmol/L (normal range, 3.6–4.8 mmol/L); chloride 108 mmol/L (normal range, 101–108 mmol/L); blood urea nitrogen 8.2 mg/dL (normal range, 8–20 mg/dL); creatinine 0.31 mg/dL (normal range, 0.46–0.79 mg/dL); total bilirubin 0.4 mg/dL (normal range, 0.4–1.5 mg/dL); albumin 4.6 g/dL (normal range, 4.1–5.1 g/dL); total protein 6.6 g/dl (normal range, 6.6–8.1 mg/dL); aspartate transaminase 32 IU/L (normal range, 13–30 IU/L); alanine transaminase 13 IU/L (normal range, 7–23 IU/L); alkaline phosphatase 1086 IU/L (normal range, 106–322 IU/L); lactate dehydrogenase 264 U/L (normal range, 124–222 U/L); and C-reactive protein 0.07 mg/dl (normal range, 0.00–0.14 mg/dl). Test results for antibodies to hepatitis B virus surface antigen, hepatitis C virus antibodies, and *Treponema pallidum* antibodies were negative. Urine analysis revealed no abnormal findings. The NWTS-5 relapse protocol involved 12 Gy of radiation therapy in 8 daily fractions and NWTS-5 relapse protocol regimen chemotherapy, including dactinomycin, vincristine, and doxorubicin [7].

### Radiotherapy treatment planning and delivery

A Vac-Lok™ (CIVCO Radiotherapy, Kalona, USA) was used to immobilize our patient. Her arms were placed on the sides away from her body or alternatively above her head by using a wing board. Appropriate sedation was used to keep her stable during the treatment. In a simulation, CT slices 2 mm in thickness were obtained from the mandible to the pelvic brim. The first CT scan was for VMAT planning with heterogeneity corrections but no gating devices. The lung clinical target volume was the entire three-dimensional bilateral lung volume. The second CT scan was a four-dimensional gated scan using the AZ-733 V respiratory gating system (Anzai Medical Systems, Tokyo, Japan). This four-dimensional scan was analyzed for maximum expansion of the lungs in the superoinferior, anteroposterior (AP), and mediolateral dimensions. The lung internal target volume (ITV) was the maximum lung expansion volume defined as the minimum intensity projection bilateral lung volume on four-dimensional CT simulation scans, including lung expansions into the anterior and posterior costophrenic recesses and bilateral hila. The lung planning target volume (PTV) was obtained by a 1-cm expansion of this ITV in all dimensions, but not outside our patient. Then, the PTV was expanded to include the entire vertebrae and mediastinum. The region of the mediastinum included lymph nodes from the sternal manubrium up to 1.5-cm inferior to the carina.

The daily fraction dose was 1.5 Gy. The total prescription dose was 12 Gy. The VMAT plan was generated by using a Monaco 5.11 (Elekta, Stockholm, Sweden) treatment planning system with the Monte Carlo algorithm. The calculation grid was set at 2 mm. The goal was that ≥ 95% of the PTV should receive ≥ 95% of the prescribed dose and that ≤ 2% and ≤ 1%, respectively, of the PTV should receive > 105% or > 110% of the prescribed dose. Heart dose–volume constraints for IMRT planning were derived from the Northwestern dosimetry study [8]. Briefly, the dose–volume constraints for the heart were as follows: doses to 20%, 40%, 60%, 80%, and 100% of the heart were 11.8 Gy, 11 Gy, 10 Gy, 8 Gy, and 4.5 Gy, respectively. For the organs at risk (OAR) doses, the maximum doses to the spinal cord, heart, and liver should be < 107%, < 110%, and < 110%, respectively. We attempted to give a more homogeneous dose to the vertebral body to prevent differential growth of her spine.

**Fig. 2:**
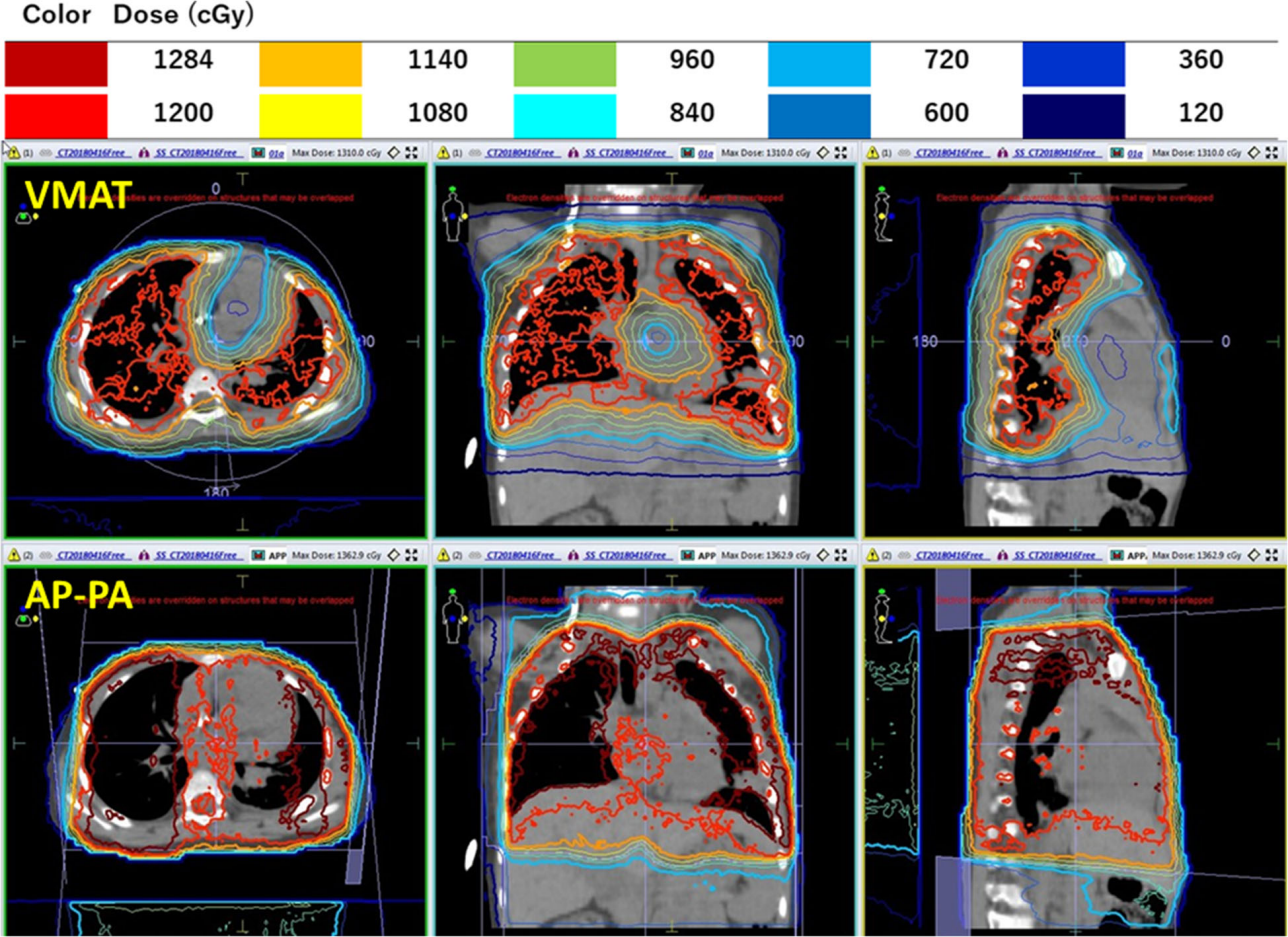
Dose distribution of the two different techniques studied. The *top panel* shows the volumetric modulated arc therapy plan and the *bottom panel* shows the standard anteroposterior–posteroanterior plan. *AP–PA* anteroposterior–posteroanterior, *VMAT* volumetric modulated arc therapy

Treatment was planned with full 360° arcs of VMAT, shown in Fig. 2. A total of 228 segments and 636 monitor units were necessary to deliver the prescribed dose to the PTV. To compare with VMAT, we calculated treatment plans that would use the standard AP–PA field technique. The AP–PA field treatment planning consisted of two equally weighted fields using 6-MV photons, and ≥ 95% of the PTV should receive ≥ 95% of the prescribed dose. The radiation doses to the lung PTV, heart, liver, and thyroid were analyzed and compared. For the PTV, the homogeneity index (HI) was used as a comparison metric for the VMAT and standard AP–PA plans. HI was defined as (D 2% − D 98%) / D 50%, where D 2%, D 98%, and D 50% indicate the doses received by 2%, 98%, and 50% of the volume, respectively.

**Fig. 3:**
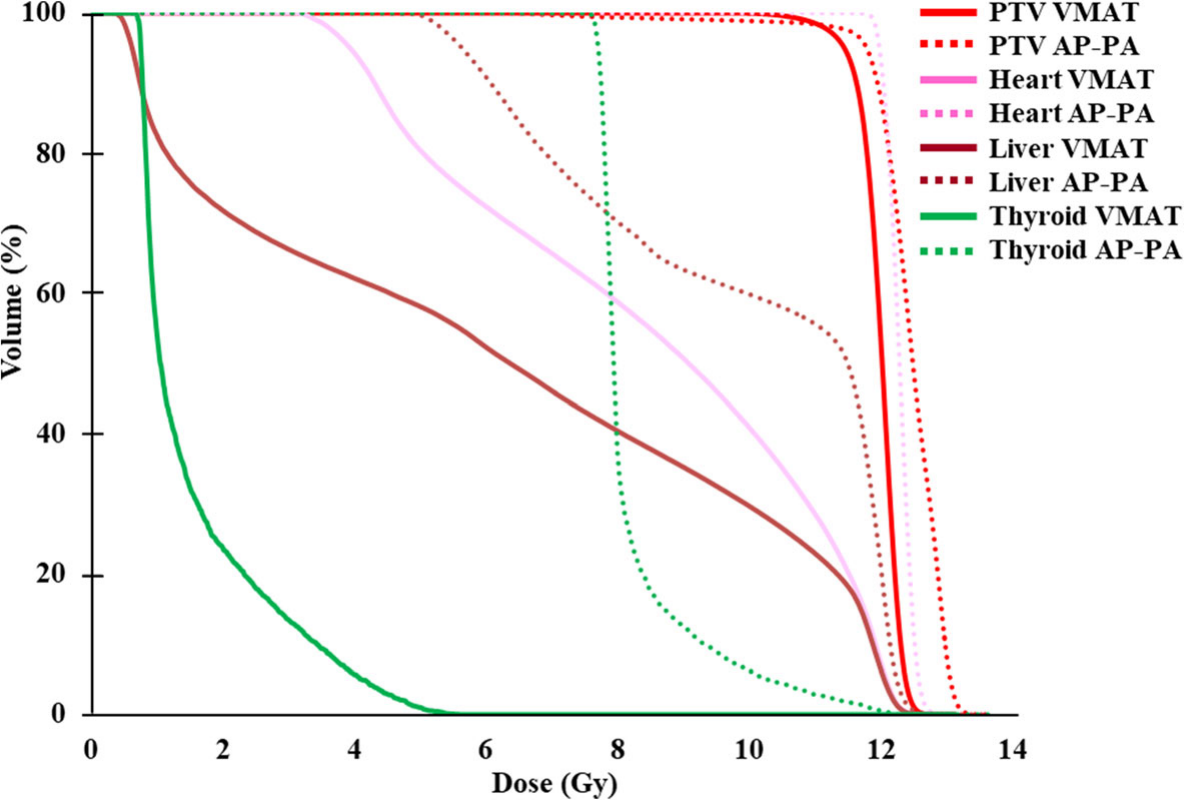
Dose–volume histograms of a patient for the two different techniques studied. The volumetric modulated arc therapy plan is represented by the *solid line* and the anteroposterior–posteroanterior plan is represented by the *dashed line. AP–PA* anteroposterior–posteroanterior, *PTV* planning target volume, *VMAT* volumetric modulated arc therapy

Figure 2 shows the dose distributions of the two different techniques studied (VMAT and AP–PA). The primary goal was that ≥ 95% of the PTV should receive ≥ 95% of the prescribed dose. The PTV coverage between the VMAT and standard AP–PA field techniques were similar. The mean heart dose was 8.5 Gy for VMAT and 12.3 Gy for the standard AP–PA field technique. The VMAT doses to the whole heart were 13.0%, 28.3%, 40.7%, 58.7%, and 86.5% for cardiac volumes (V)11.8, V11, V10, V8, and V4.5, respectively. Those heart doses were almost 100% of the standard AP–PA field techniques. The mean liver doses were 6.2 Gy for VMAT and 9.9 Gy for the AP–PA field technique. The mean thyroid doses were 1.6 Gy for VMAT and 8.2 Gy for the AP–PA field technique. Table 1 summarizes the details of the dose statistics for the PTV coverage, heart, liver, and thyroid gland. We confirmed the dosimetric advantages of VMAT over the standard AP–PA field WLI technique, including superior cardiac protection and superior dose uniformity in the lungs with fewer hot spots (Fig. 2). These findings are illustrated in the dose–volume histogram in Fig. 3.

**Table 1:** Dose statistics comparison

Structure	VMAT	AP–PA
PTV		
D98 (Gy)	11.1	11.3
D95 (Gy)	11.4	11.8
D2 (Gy)	12.4	13.1
Heart		
V4.5 (%)	86.5	100.0
V8 (%)	58.7	100.0
V10 (%)	40.7	100.0
V11 (%)	28.3	100.0
V11.8 (%)	13.0	99.8
Mean (Gy)	8.5	12.3
Liver		
Mean (Gy)	6.2	9.9
Thyroid gland		
Mean (Gy)	1.6	8.2
Homogeneity index		
	0.111	0.147

*AP–PA* anteroposterior–posteroanterior field, *PTV* planning target volume, *VMAT* volumetric modulated arc therapy

### Quality assurance of the treatment plan

The calculated VMAT plan was verified prior to treatment by using the ArcCHECK (Sun Nuclear, Melbourne, USA). A passing rate of 100.0 was achieved (3.0-mm distance-to-agreement; 3.0% dose difference with reference to the maximum dose of the calculated volume; doses < 10.0% of the normalization dose were not included in the analysis). Additional measurements with ionization chambers were made by using a RT-3000 Phantom (R-Tech, Tokyo, Japan). An ionizing chamber PTW31014 (PTW-Freiburg, Freiburg, Germany) was used to measure doses at three points. The measured values were compared with the values in the treatment planning system. The dose deviations were all < 3%. VMAT irradiation was delivered by using 6-MV photon beams from a Synergy® S linear accelerator with the Agility™ collimator (Elekta, Stockholm, Sweden). Daily cone-beam CT was carried out to verify correct positioning.

### Clinical course

Our patient tolerated radiotherapy well and completed the course as planned. Toxicity was recorded daily during radiotherapy and monthly thereafter. One month after the completion of radiotherapy, our patient did not complain of symptoms attributable to radiation. Routine follow-up CT scans obtained 1 year after radiotherapy did not show appearance of lung metastases. No late toxicities were observed at the latest follow-up, 13 months after completion of radiotherapy.

## Discussion

We presented a 3-year-old girl with lung metastases who received WLI using VMAT. We confirmed the dosimetric advantages of VMAT over the traditional AP–PA field technique, including superior cardiac protection and superior dose uniformity in the lungs. To the best of our knowledge, this is the second case treated with WLI using VMAT.

Dramatic improvements in survival have been achieved for children and adolescents with cancer [9]. Now that long-term survival cases can be expected, late adverse-event alleviation has become more important than it was previously. Late adverse events of cardiovascular disease, after recurrence of the original cancer and development of second primary cancers, have been reported to be the leading cause of premature mortality among long-term survivors of childhood cancer [10–14]. Various studies have demonstrated a substantially increased risk of heart failure, pericardial disease, and valvular disease with higher radiation doses [15–20]. Pein *et al.* reported a 6.48-fold risk for cardiac abnormalities in children receiving mean heart doses in the range of 5–20 Gy [21]. Tukenova *et al*. assessed a total of 4122 5-year survivors of childhood cancer and found that the risk of dying as a result of cardiac diseases was significantly higher in the individuals who received an average radiation dose that exceeded 5 Gy to the heart [12]. Green *et al.* estimated that the risk of congestive heart failure would increase by a factor of 1.6 for every 10 Gy increase in lung radiation [22]. In addition, radiation to the chest for lung metastases may also result in thyroid disease and portal hypertension [23]. Therefore, radiation oncologists must make efforts to reduce the irradiated doses to normal organs as much as possible.

Recent progress in radiotherapy has been reported to substantially reduce heart doses by IMRT even in WLI [24]. In a study that performed WLI using IMRT by Kalapurakal *et al.* [24] with different mean prescribed V, the percentage of prescribed radiation doses were 96%, 85%, 65%, and 39% to the target volumes of the heart of V50, V67, V83, and V95, respectively. Comparing their results with those of our study, we used VMAT with a prescribed dose of 12 Gy to achieve 72%, 58%, 41%, and 22% to the target heart volumes of V50, V67, V83, and V95, respectively. The technique developed by Kalapurakal *et al.* [24] differs from ours mainly in two ways: (1) only fixed-field IMRT techniques, such as “step and shoot” and “sliding window,” were allowed, whereas we used VMAT; and (2) the whole lung IMRT dose consisted of 15 Gy in 10 fractions (14 patients) and 12 Gy in 8 fractions (6 patients), whereas we delivered 12 Gy in 8 fractions to our patient. These factors most probably resulted in better heart sparing in our patient than that achieved by Kalapurakal *et al.* [24].

Comparisons of VMAT and fixed-field IMRT have been evaluated for a large number of tumor sites, and these studies have largely demonstrated that VMAT is capable of creating analogous or better dose distributions [25–29]. In addition, VMAT has the extra benefit of more rapid treatment time [5]. Increased treatment time in the management of childhood cancer has more than a few undesirable implications, including requiring patients to spend long periods on the radiotherapy couch, which can lead to patient distress and increases the risk of movement of the patient. Therefore, VMAT may be a desirable technique for treating childhood cancer. To the best of our knowledge, the only case involving WLI using VMAT was in a study of Papachristofilou *et al*. [6]. Their case received a non-standard dose of 18 Gy of WLI. Our case report, which involved a prescribed standard dose of 12 Gy, will provide more useful clinical information than provided in the previous report. Our results demonstrated the feasibility of the VMAT technique in the treatment of WLI. The radiation delivered by using the VMAT technique was well tolerated without treatment-related toxicities during and after treatment.

This case report had some limitations that should be considered. First, this is a report of a single patient with a short follow-up after VMAT. A prospective study with a large number of patients and longer follow-up should be conducted to validate our results by using standardized procedures. Second, low-dose leakage in normal tissues, which tends to increase with IMRT and VMAT, may increase the risk of secondary malignancies. Further follow-up of patients treated by this technique is necessary to quantify this risk.

## Conclusion

In conclusion, our experience with this patient demonstrated that effective heart dose sparing was possible for WLI using VMAT and should be considered by radiologists for use in their practices.

## Data Availability

The cancer registry dataset can only be acquired with permission of the Kyoto Prefectural University of Medicine. Therefore, we cannot release the clinical data for this study.

## Abbreviations

AP: Anteroposterior
AP–PA: Anteroposterior–posteroanterior
CT: Computed tomography
EFS: Event-free survival
HI: Homogeneity index
IMRT: Intensity-modulated radiation therapy
ITV: Internal target volume
NWTS: National Wilms Tumor Study Group
OAR: Organs at risk
OS: Overall survival
PDA: Patent ductus arteriosus
PTV: Planning target volume
VMAT: Volumetric modulated arc therapy
WLI: Whole lung irradiation
WT: Wilms’ tumor
